# *Trans*-regulatory loci shape natural variation of gene expression plasticity in *Arabidopsis*

**DOI:** 10.1093/genetics/iyaf116

**Published:** 2025-06-19

**Authors:** Mariele Lensink, Grey Monroe, Daniel J Kliebenstein

**Affiliations:** Department of Plant Sciences, University of California Davis, Davis, CA 95616, USA; Integrative Genetics and Genomics Graduate Group, University of California Davis, Davis, CA 95616, USA; Department of Plant Sciences, University of California Davis, Davis, CA 95616, USA; Department of Plant Sciences, University of California Davis, Davis, CA 95616, USA

**Keywords:** A*rabidopsis*, quantitative traits, gene expression, salicylic acid, recombinant inbred lines, eQTL, *trans*-regulation

## Abstract

Organisms regulate gene expression in response to environmental cues, a process known as plasticity, to adjust to changing environments. Research into natural variation and the evolution of plasticity frequently studies *cis*-regulatory elements with theory suggesting they are more important evolutionarily than *trans*-regulatory elements. Genome-wide association (GWA) studies have supported this idea, observing a predominance of *cis*-loci affecting plasticity. However, studies in structured populations provide a contrasting image, raising questions about the genetic architecture of natural variation in plasticity. To circumvent potential statistical difficulties present in GWA studies, we mapped loci underlying transcriptomic plasticity in response to salicylic acid (SA) using recombinant inbred lines generated from 2 random *Arabidopsis thaliana* accessions. We detected extensive transgressive segregation in the SA response, suggesting that plasticity to salicylate in *Arabidopsis* is polygenic. Most loci (>75%) underlying this variation act in *trans*, especially for loci influencing plasticity. *Trans*-acting loci were enriched in genome hotspots, with predominantly small-effect sizes distributed across many genes. This could potentially explain their under-discovery in GWA studies. This work reveals a potentially important role for *trans*-acting loci in plastic expression responses, with implications for understanding plant adaptation to different environments.

## Introduction

Plasticity can be defined as the capacity of an individual genotype to change a trait in response to external stimuli (Pigliucci *et al.* 2006). Plastic responses allow organisms to optimize their phenotypes to respond to and persist in their environment. As such, plasticity can be evolutionarily advantageous by enabling a species to persevere at a new phenotypic optimum until genetic variation arises that generates a more adaptive phenotype. This model largely depends on the strength of selection and the time scale of evolutionary change (Ghalambor *et al.* 2007; Levis and Pfennig 2016). Furthermore, genetic variation in plasticity has been shown to be advantageous in novel environments and increase the likelihood of evolutionary rescue (Walter *et al.* 2023). Understanding how genetic variation in plasticity is genomically distributed will provide valuable insight into the regulation of response to environmental stimuli and inform strategies to increase plant resilience to changing climates.

While plasticity affects a wide array of physiological and morphological traits, gene expression is a key tool for studying how plasticity evolves and varies. Gene expression is inherently dynamic, considered to be more directly linked to genetic changes than morphological phenotypes, and relatively easy to quantify en masse with transcriptomics (Schlichting and Smith 2002; de Nadal *et al.* 2011). Thus, studies on a gene's expression plasticity can yield more detailed mechanistic insight into how plasticity variation occurs than may be possible with a physiological or morphological trait. These benefits have led many studies to use transcriptomics to measure and map plasticity in naturally variable populations. The identified genomic loci that explain variation in transcription are called expression quantitative trait loci, or eQTL. In the context of a single gene, plasticity can be *cis*-regulated by genomic elements adjacent to or within the gene of interest, or *trans*-regulated by loci unlinked to the gene of interest. Natural variation in plasticity is theorized to be predominantly controlled by *cis*-regulators, as *cis* effects typically have less pleiotropic effects, are predicted to experience relaxed or balancing selection (as opposed to purifying selection for *trans*-regulators), and are more influential in gene expression divergence (Hoekstra and Coyne 2007; Metzger *et al.* 2017; Brown and Kelly 2022; Vande Zande *et al.* 2022). In contrast, *trans*-regulatory mutations tend to be more pleiotropic with potentially deleterious effects. The resulting theory that *cis*-acting loci are under weaker purifying selection than *trans*-acting eQTL is supported by genome-wide association (GWA) studies of natural populations that identify *cis*-eQTLs as the primary identifiable basis for plasticity variation (Mai *et al.* 2021; Sun *et al.* 2023; Xu *et al.* 2023). Consequently, research regarding the natural variation in plasticity and its evolution often focuses on *cis*-acting changes (Massouras *et al.* 2012; He *et al.* 2016, 2021; Pruunsild and Bading 2019; Dardiry *et al.* 2023; Takou *et al.* 2024).

In contrast to GWA populations, many studies using structured populations [e.g. recombinant inbred lines (RILs) originating from a biparental cross] find mainly *trans*-acting regulation of gene expression and plasticity. In recombinant inbred strains of *Caenorhabditis elegans* and *Zea mays*, a significantly larger proportion of *trans*-acting genes had plasticity eQTL in contrast to *cis*-acting genes (Li *et al.* 2006, 2013). In inbred mice, expression plasticity was also largely attributed to *trans*-acting loci (Ballinger *et al.* 2023). Supporting these observations was work on a small *Arabidopsis thaliana* (*Arabidopsis* hereafter) collection of accessions that showed the majority of plasticity variation links to coexpressed gene networks, suggesting mainly *trans*-eQTL (van Leeuwen *et al.* 2007). The difference between GWA and biparental populations establishes a discord regarding whether variation in gene expression plasticity is predominantly controlled by *cis* or *trans* factors.

One possible explanation for the difference in the relative proportions of *trans* and *cis*-acting eQTL between structured and GWA populations is ascertainment bias, caused by the nature of *cis* vs *trans* gene regulation. *Cis*-eQTL frequently has larger effects on a transcript's expression compared with *trans* loci. GWA studies, leveraging the diversity of alleles in natural populations, are generally powered to detect loci with moderate to large effects but are less effective at detecting loci with smaller effects. This inherent bias in GWA studies favors associations with moderate-frequency alleles that exhibit large effects (Myles *et al.* 2009; Sham and Purcell 2014), and detected rare alleles are likely to have an overestimated effect size (Göring *et al.* 2001). In selfing species such as *Arabidopsis*, population structure and the trait of interest are more likely to be confounded, complicating the detection of true associations in GWA studies (Zhao *et al.* 2007). In contrast, structured populations, by having fewer genotypes per locus and balanced allele frequencies, are better suited to find these smaller effect loci (Gibson 2012; Korte and Farlow 2013; Marigorta *et al.* 2018; Liu *et al.* 2019). The advantages and disadvantages of GWA and QTL studies are comprehensively discussed in Josephs *et al.* (2017).

Previous work in *Arabidopsis* has followed this trend; GWA of transcript expression in specific conditions and their plastic response has largely found and focused on *cis*-regulation (Zhang *et al.* 2011; Lasky *et al.* 2014; He *et al.* 2016, 2021; Zan *et al.* 2016). To map the architecture of plasticity and assess the relative contributions of *cis-* and *trans*-eQTL within *Arabidopsis*, we used the structured Bayreuth (Bay-0) × Shahdara (Sha) *Arabidopsis* RIL population. This RIL population was treated with the defense signaling hormone, salicylic acid (SA), or received control treatment, and the transcriptome was measured at 28 h post-treatment.

SA was selected for plasticity analysis because it is a key hormone regulating plant defense, and its exogenous application induces responses that mirror endogenous signaling during pathogen attack. In *Arabidopsis*, SA is biosynthesized by the isochorismate pathway involving ICS1, EDS5, PBS3, and EPS1 (Peng *et al.* 2021). SA then interacts with NPR1 and/or NPR3/4 to regulate sets of targets, including TGACG-Binding transcription factors, typically by Sumoylation or Ubiquitination of the targets (Zhang and Li 2019; Peng *et al.* 2021). The ability to synthesize and respond to SA is highly conserved across land plants and essential for defense against pathogens (Spoel and Dong 2024). SA is also highly interconnected with other defense pathways, such as the jasmonic acid pathway, creating numerous feedback and feedforward loops that can regulate its basal and response behavior (Peng *et al.* 2021; Spoel and Dong 2024). While SA-mediated defense responses are highly conserved across land plants, the specific biosynthetic and regulatory pathways can vary across plant families (Peng *et al.* 2021). In this way, SA treatment provides a defined treatment that can induce plasticity responses similar to those in plant-pathogen interactions while minimizing physiological shifts. It should be noted that, because SA is an endogenous plant phytohormone, it exists in small quantities in untreated plants. As a result, our control group does not explicitly represent an absence of signal. The Bay-0 × Sha population was chosen as these are diverse representatives of *Arabidopsis* with species typical SA responses (West *et al.* 2006, 2007; van Leeuwen *et al.* 2007). The 28-h time point was chosen to maximize the plasticity response of gene expression while minimizing any physiological change (van Leeuwen *et al.* 2007). Using this transcriptome data, we mapped plasticity eQTL controlling variation in the SA response and showed that they predominantly function in *trans* and with small effects.

## Materials and methods

### Experimental design and data collection

We acquired transcript abundance data for the Bay-0 × Sha parental and F8 RIL lines (Loudet *et al.* 2002) from a previous microarray experiment (West *et al.* 2007). In this experiment, the parents and 211 RILs were grown in a randomized design and treated with either a control silwet L77 (a surfactant), or 0.3 mM SA in silwet L77 (West *et al.* 2007). The control and SA-treated plants were grown together and tissue was collected 28 h after treatment (van Leeuwen *et al.* 2007). Growth and treatment protocols for plants are fully described in West *et al.* (2007). The entire experiment was independently replicated twice. The transcriptomic analysis of the SA treatment has not been previously reported or compared with the control treatment to investigate plasticity.

The microarray used was the ATH1 Genome Array where each of 24,000 transcripts is measured using 11 probes per transcript, with each probe having 25 bases (ThermoFisher Scientific). The 11 independent and non-overlapping probes per gene are predominantly in the 3′UTR of the gene and designed using the *Arabidopsis* genome sequence to maximize the ability to specifically measure an individual transcript compared with any closely related sequences. Any potential cross-hybridizing probe sets are identified in the original design. This minimizes potential cross-signal caused by related sequences for other genes (Redman *et al.* 2004).

### Transcriptome analysis

**Fig. 1.**
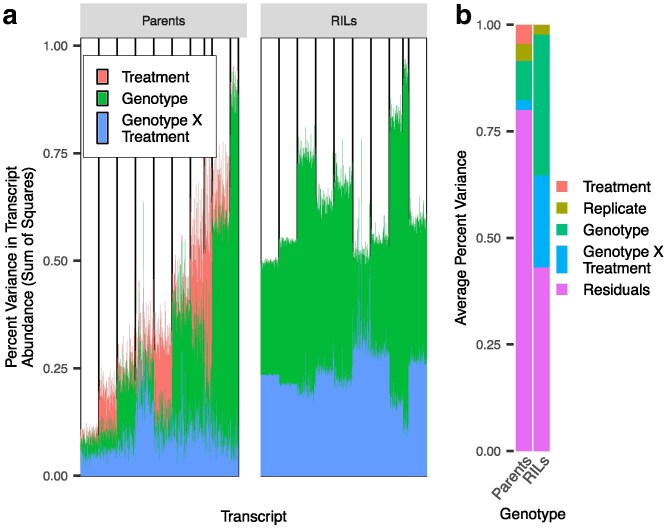
Changes in gene expression across environments are attributed to separate experimental variables between parent and RIL genotypes. a) Variance in transcript abundance (sum of squares/TSS) attributable to each experimental variable for parents and RILs. Results from the type III ANOVA for each transcript were clustered into 10 variance profiles using *k* means, and 1,000 transcripts were sampled from each cluster for visualization. b) Average proportion of variation (sum of squares) across transcripts explained by each experimental variable.

Using the expression estimate for each transcript across all the genotypes, we tested the distribution of variance linked to experimental replicates, treatment, genotype, and their interaction in both the parental accessions and RILs. This was done using a linear mixed model within the R package “R/car” (Fox and Weisberg 2018), with experimental replicate as a random effect while genotype and treatment were modeled as fixed effects. The sum of squares for each factor for each transcript was calculated using a type III ANOVA. These variance results were then clustered into 10 profiles using *k*means to better visualize the major trends in variation in gene expression attributed to the model's effects. Samples of 1,000 transcripts from each cluster were randomly selected and plotted, independently in parents and RILs (Fig. 1a).

The average expression for each transcript within each treatment was estimated by calculating the mean value of every transcript for every individual across biological replicates. The mean values were then used to visualize how the transcriptome varies across recombinant inbred and parental lines via a principal component analysis (PCA) using R/stats. This was repeated after removing transcripts that contained *cis*-eQTLs to test if the pattern was altered.

### eQTL analysis

The average value per transcript per treatment per genotype was then used for eQTL mapping. The RILs were previously genotyped using a combination of genetic markers and the map was obtained from (Loudet *et al.* 2002; West *et al.* 2006). Using this map, which has an average marker distance of 0.17 cM, we conducted eQTL analyses with 210 of the 211 RILs due to missing genotype information for RIL 417. For this analysis, we used the R/QTL2 package using Haley–Knott regression (Broman *et al.* 2019). eQTL analysis was performed on transcript values obtained using either silwet (control) or SA samples and as a mathematically derived value called “delta.” Delta represents an estimate of the plasticity phenotype obtained by calculating the difference in each transcript abundance across the 2 treatments divided by the average transcript abundance across the 2 treatments: (SA-control)/[(SA + control)/2]. This was calculated independently for each genotype for each transcript. These 3 eQTL analyses, being SA, control, and delta, will hereafter be referred to as “expression phenotypes.” QTL locations were estimated for each transcript for each expression phenotype within R/QTL2 using a significance threshold of 2 LOD. This threshold was previously shown by permutations to be a suitable balance of type I and type II error rates (West *et al.* 2006, 2007).

The distribution of eQTLs across the genome was plotted using a sliding window with a 10 cM range and a step size of 1, in accordance with West *et al.* (2007). For the sake of downstream analysis, transcript names were converted to the standard *Arabidopsis* Genome Initiative naming system. Changes in genome predictions and gene annotations led to the removal of 1,201 genes that were measured by the microarray but are not present in the current genome annotation model. This left 88,729 eQTLs. To identify potential eQTL hotspots, permutation analysis was used to estimate a global significance threshold for QTL position within each expression phenotype (control, SA, and delta). The 95th percentile value was used for the threshold to indicate significantly enriched eQTL hotspot regions.

### Determining *cis-* vs *trans*-eQTL position and treatment conditionality

To determine *cis*-acting vs *trans*-acting QTL, the position of every gene in the *A. thaliana* genome was interpolated into the genetic map using the Single Feature Polymorphism marker positions from West *et al.* (2007). The threshold for calling an eQTL *cis* was if the eQTL for a transcript was located 10 cM in either direction of the transcript's genomic position based on which was the more conservative window size in favor of calling *cis-*eQTL according to West *et al.* (2007). To assess an eQTLs treatment effect, we compared the map positions for eQTLs for a transcript across the control, SA, and delta expression phenotypes. If there was an eQTL for a transcript in 1 or more of these conditions that mapped within 5.5 cM, it was considered to be the same QTL. The increased distance of 5.5 cM was chosen as a more conservative value for this measure to account for the small population size, which limits the recombination and resolution of the genomic map. The eQTL was then collapsed, and the average position was recorded as the shared map position for further analysis. Previous work has shown that the presence of SNPs within the probes for the different transcripts on the microarray had minimal impact on the identification of eQTLs (West *et al.* 2006, 2007; van Leeuwen *et al.* 2007; Chan *et al.* 2010).

### Calculating eQTL effect size

The effect size for each eQTL for each transcript was estimated using linear regression analysis for which the predictor variable for each model was the genotype of a given eQTL across the RILs, and the response variable was the transcript abundance across the same RILs within a specific expression phenotype (control, SA or delta). The *R*-squared value ascribed to the genotype from each model was then used to represent the effect size of each eQTL for each transcript in each expression phenotype (control, SA, or delta).

### Sequence comparison of Bay-0 and Sha to explore potential mechanistic origins of eQTLs

To explore the molecular mechanisms that might influence the presence and magnitude of eQTLs, we used the TAIR10 *Arabidopsis* Col-0 reference genome, along with Bay-0 and Shahdara assemblies from Lian *et al.* (2024). We analyzed synteny-informed gene copy number variations using GENESPACE (Lovell *et al.* 2022). For each *Arabidopsis* accession, we quantified the number of paralogs associated with transcripts exhibiting eQTLs across different expression phenotypes. An eQTL was considered to be located in a hotspot if it fell within 5 cM of the salicylate or jasmonate hotspot regions on chromosomes 2 and 5, respectively (refer to Supplementary Table 1). Due to differing gene nomenclature in the Bay-0 and Shahdara assemblies, we used the Columbia reference to identify potential *trans*-eQTLs caused by the presence or absence of paralogs within 10 cM of the eQTL loci. Additionally, we excluded 1,000 control eQTL transcripts from further comparative analysis after discovering they were fragments not representing whole genes in the TAIR assembly. The location of paralogs in centimorgans was then inferred using the same genetic map as previously described.

## Results

### Increased influence of genotype and plasticity on transcription in RILs

To understand the genetic and environmental factors controlling transcriptional variation, we modeled the relative contributions of genetics, treatment (plasticity/environment as the SA vs control application), and the interaction of genetics and treatment (i.e. genotype × environment/plasticity) to variation in transcript abundance. This was done independently within the Parents and RIL genotypes across transcripts. In this analysis, variation between the control and SA treatments is an estimate of the plastic response to SA treatment. For each transcript, we performed linear modeling and used the results to calculate the proportion of variance for each factor. We found distinct differences between the parents and RILs in the relative contributions of genotype, treatment, and genotype × treatment in influencing the expression of transcripts. First, most of the transcript variance in the RILs is associated with genotype and genotype-treatment while the parents' transcript variance is predominantly in the treatment term (Fig. 1a and b). The transcripts in the parents exhibit diverse expression patterns, with some being entirely genotype-dependent, others predominantly plasticity-dependent (the difference between SA and control), and some having no contribution from any of the experimental components. The parents also exhibit higher variability in the contribution of residuals to expression than the RIL population (Fig. 1a). In contrast to these variable contributions in the parents, the same transcripts in the RILs were all influenced by genotype and genotype by treatment at similar levels (Fig. 1a). The shift where some transcripts in the parents are mainly influenced by treatment to the same transcripts being mainly influenced by genotypes × treatment in the RILs suggests the potential for multiple polymorphic loci with opposing effect directions influencing SA response in the parents. Upon crossing and recombination, it is possible that the alleles at these loci shuffle to create new combinations in the RILs. If this is true, there should be transgressive segregation in SA responses (plasticity) in the RILs where the RILs have SA responses not found in the parental accessions.

### Greater transcriptome variation in RILs

**Fig. 2.**
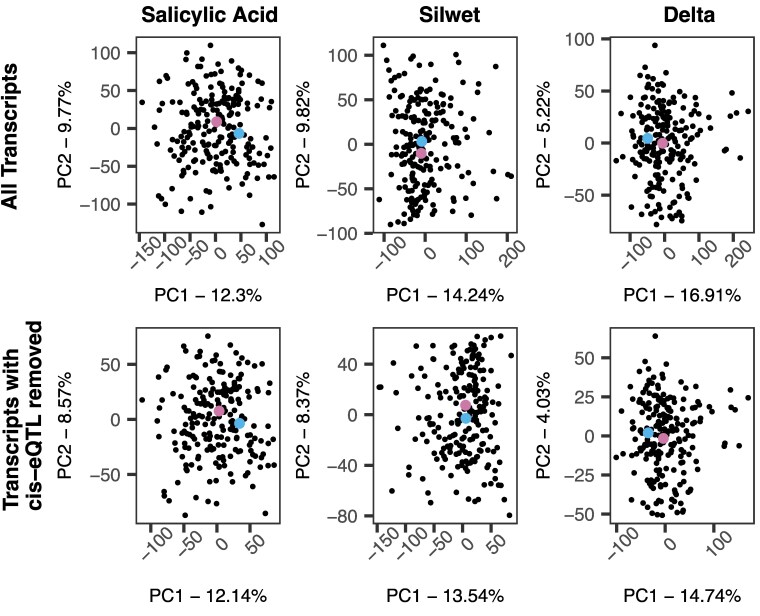
Transcriptome variation across recombinant inbred and parent lines. PCA reveals high variation in transcript abundance in RILs compared with parental lines in Bay-0 (blue) and Sha (pink). Analysis using the entire transcriptome is shown on the top row while transcriptomes without genes containing *cis*-eQTL is shown on the bottom row.

**Fig. 3.**
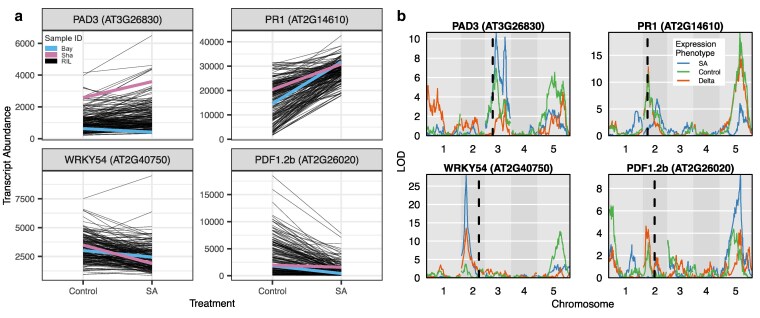
The RIL mean expression and plasticity response of classic SA response genes exceeds the range set by parent lines. a) Transcript abundance of marker genes involved in SA responses across environmental treatment groups. Lines represent abundance in individual RILs (black) and the parent lines Bay-0 (blue) and Shahdara (pink). b) The panels to the right show the corresponding eQTL map for each transcript for each expression phenotype (SA treatment in blue, control in green, and delta in orange).

In light of the differences in how genotype and plasticity influence transcripts between the parents and RILs (delta of SA response), we analyzed the overall pattern of individual transcriptomes across RILs and parents to understand general trends better. We performed PCA across all individuals first using their whole transcriptome. Results from this PCA are consistent with the idea that there is transgressive segregation for the transcriptomes within the RILs. For SA and silwet treatments, along with delta (representing a change in expression across the environment, or plasticity), the RILs exhibited more transcriptome diversity than the parent lines (Fig. 2). This pattern implies that the generation of new genotypes also generates new expression patterns that are not present within the parental genotypes. To further visualize this, we queried 4 individual transcripts classically associated with SA responses. These 4 are commonly used genes across the literature that act as biomarkers for measuring response to SA. As such, we chose them to illustrate how expression can vary across treatment between the parental accessions and the RILs. PR1 and WRKY54 are downstream response genes typically upregulated in response to SA (Wang *et al.* 2006; Vlot *et al.* 2009). PDF1.2b produces an antimicrobial peptide in response to jasmonate, but is generally expected to be suppressed by salicylate (Manners *et al.* 1998). Finally, PAD3 is involved in camalexin biosynthesis and is expressed combinatorially with salicylate, jasmonate, and other signals (Ferrari *et al.* 2007). Plotting each gene's change in expression in response to treatment across individuals showed that for each of these genes, the plasticity responses of the RILs exceed the range set by the parents (Fig. 3), which is not unexpected in segregating hybrid populations (Rieseberg *et al.* 1999). Interestingly, this led to shifts in the sign and magnitude of SA response across RILs with genes typically induced by SA showing no change or even being repressed (Fig. 3). The increased variation in the global transcriptome and for expression response of known SA response genes between RILs and parental lines is evidence for transgressive segregation for gene expression plasticity within this population. Directly mapping loci and identifying opposing allelic effects between loci can validate this model.

### *Trans*-regulation as a major driver of gene expression plasticity

**Fig. 4.**
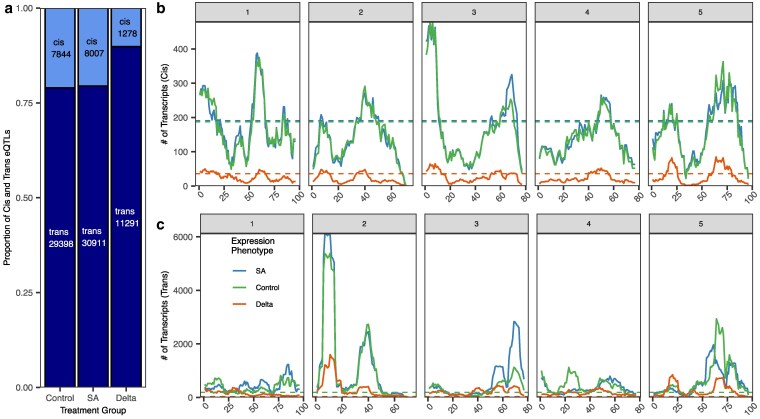
Quantification and position of *cis-* and *trans*-eQTLs across treatment groups. a) The proportion of detected eQTL mapping to *cis* (dark gray) or *trans* (light gray) positions for each treatment group. b) Number of eQTLs at each position (cM) across all 5 chromosomes of the *A. thaliana* genome for *trans* b) and *cis* c) eQTL. The dotted lines represent the 95th percentile value of 1,000 randomized permutations for each treatment group. The largest hot spots for all 3 groups are on chromosome 2. The chromosomes end at positions 100 cM for I, 70 cM for II, 80 cM for III, 80 cM for IV, and 100 cM for V.

To identify the genetic loci involved in the regulation of gene expression in the context of both environment-specific expression and plasticity, we conducted 3 quantitative trait loci analyses on the following expression phenotypes for each transcript: expression in SA, silwet (control), and delta. We mapped eQTL for each expression phenotype and used the identified eQTL to quantify *cis*- and *trans*-regulatory eQTLS and map hotspot positions. In total, we detected 8,007 *cis* and 30,911 *trans*-eQTLs for SA, 7,844 *cis* and 29,398 *trans*-eQTLs for control, and 1,278 *cis* and 11,291 *trans*-eQTLs for delta. There was an average of 1.54 *trans*-eQTL per transcript and 0.70 *cis*-eQTL per transcript across all 3 expression phenotypes. For all expression phenotypes, most eQTL were *trans*-acting (∼80%) with a slightly higher fraction in the delta phenotype (90%), indicating that regulation of plasticity is highly polygenic (Fig. 4a).

The *trans*-eQTL for all 3 expression phenotypes (SA, control, and delta) display similar distributions across the genome, albeit at different magnitudes. The major hotspots are located on chromosomes 2, 3, and 5, although there are secondary hotspots existing on every chromosome (Fig. 4b). This general pattern confirms the previous eQTL map (West *et al.* 2007), performed on the same control expression data. In contrast, the distribution of *cis*-eQTLs does not display any hotspot and follows the pattern of gene density across the genome. This co-localization of eQTL hotspots across the expression phenotypes implies a shared genetic architecture between plasticity (delta) and within-treatment expression variation (SA and control). It is clear that most QTLs are *trans*-acting, especially for plasticity, and are similarly distributed across the genome for the different expression phenotypes (Fig. 4). The presence of multiple hot spots for *trans*-eQTL, particularly for plasticity, indicates that multiple loci influence a large number of transcripts. Supporting the observation of transgressive segregation, these *trans* hotspots often have alleles with opposing directional effects for the same transcript (Fig. 3a). This suggests that Bay and Sha parents have variation at a set of loci controlling SA responses and that the allelic variation is balanced such that the parents have the same transcriptome responses. When the allelic variation at these loci is shuffled, the parents can have dramatically different SA responses. Given that the Bay and Sha parents were randomly chosen with respect to SA signaling, the presence of transgressive variation suggests that *Arabidopsis* accessions may hide underlying genetic variation in SA responses more broadly (Koornneef *et al.* 2008).

### Plasticity is largely controlled by *trans-*regulatory loci of small to moderate effect size

**Fig. 5.**
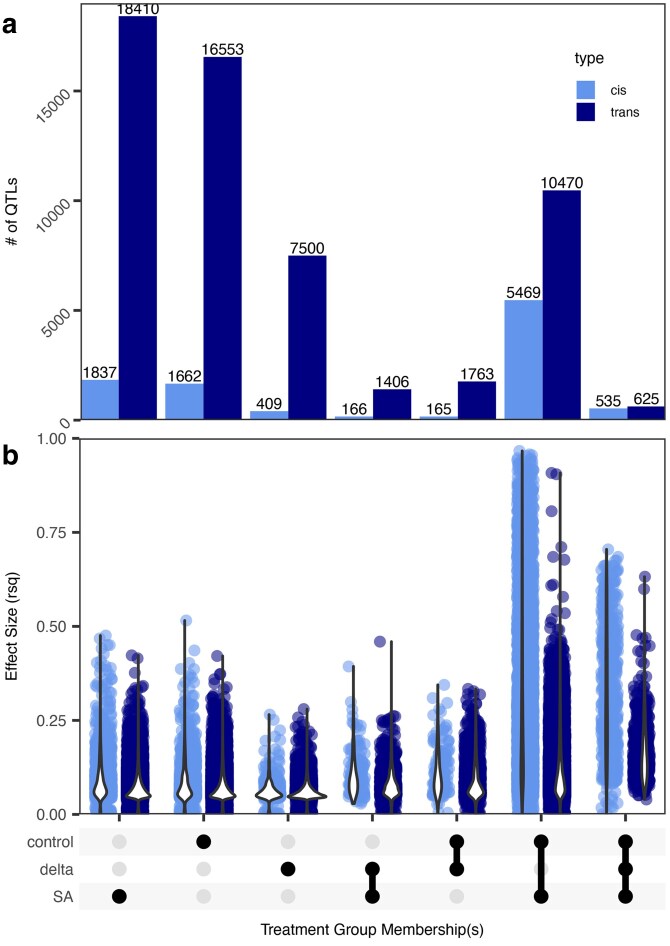
Quantity and effect size of *cis* and *trans*-eQTL according to membership across treatment groups. *Cis* (dark gray) and *trans* (light gray) eQTLs present in each of the 3 treatments. a) Number of eQTLs present in SA, silwet, delta, or a combination of treatment groups. b) Effect size of eQTLs according to treatment group membership. Effect size is represented by an *R*-squared value. In cases where an eQTL exists in more than 1 group, the mean *R*-squared value is plotted.

We sought to characterize the effect size and relative abundance of *cis* and *trans*-eQTLs existing in and across the 3 expression phenotypes (control, SA, and delta). To estimate the phenotypic effects of each eQTL, we used a linear model for every eQTL detected for each transcript in each expression phenotype. We used the proportion of variance explained (*R*-squared) by genotype to estimate the eQTL's effect size. To not double count eQTLs for transcripts, we compared eQTLs for each transcript across the 3 expression phenotypes and grouped them as a single eQTL if 2 eQTLs regulating the same transcript existed within 5.5 cM of each other across the 3 expression phenotypes. This showed that eQTLs influencing plasticity are mostly of low to moderate effect size, both when the eQTL is exclusive to plasticity response, and when the eQTL is shared across all expression phenotypes. Additionally, eQTLs that influenced expression plasticity (delta, SA-delta, control-delta, and SA-control-delta) were less frequent than eQTLs found for only the non-plasticity expression phenotypes. Finally, the plasticity-associated eQTLs had greater proportions of *trans*- to *cis*-QTL, and low effect sizes compared with eQTLs that solely affected the accumulation in SA, control, or SA-control (Fig. 5; Supplementary Fig. 3).

**Fig. 6.**
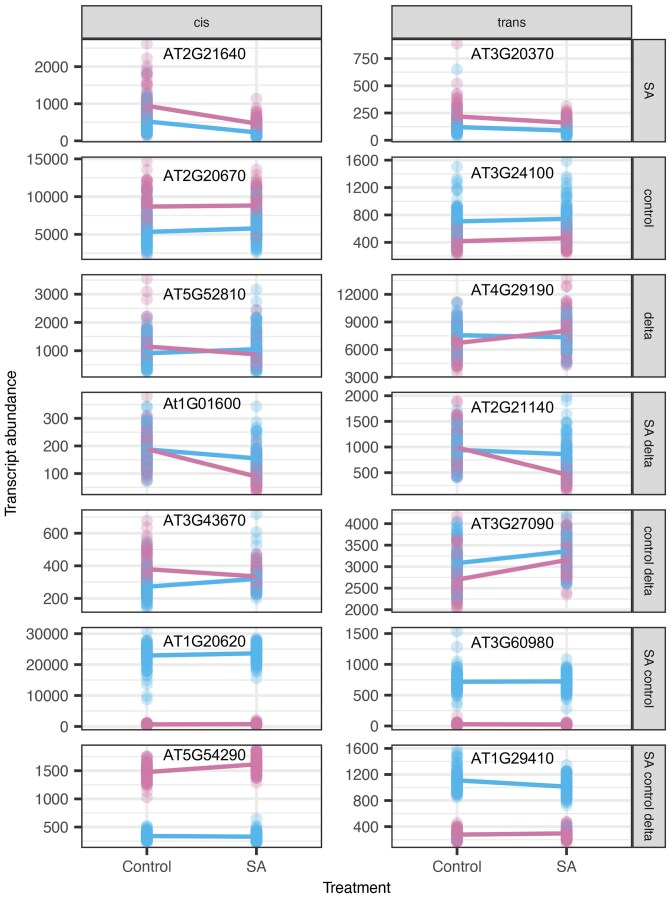
Expression across environments of high-effect size eQTL according to treatment group membership. The transcript abundance for the highest effect size eQTL across type (*cis* or *trans*) and treatment group combination was plotted. Individual genotypes are colored according to the genotype (Bayreuth in blue or Shahdara in pink) at the given eQTL. Mean expression across individuals within a given genotype is represented by a solid line.

It was initially puzzling that there were eQTL that linked to a transcript in SA but not in control, or *vice versa*, yet had no signal upon a plasticity/delta effect. However, plotting the effect of these eQTL on specific transcripts showed that they are likely a power-to-detect issue and likely have similar effects in both conditions and were under the significance threshold in the other condition (Fig. 6). In agreement with this, the eQTL effects for a given transcript showed the same magnitude and direction of change in expression across SA and control, indicating similar effects in both SA and control and that the original observation was caused by false negative eQTL. In contrast, transcript eQTLs linked to SA-delta and control-delta effects had much stronger plasticity effects with a greater divergence in the slope of the response between the 2 genotypes (Fig. 6).

### Large-effect *trans*-eQTL

**Fig. 7.**
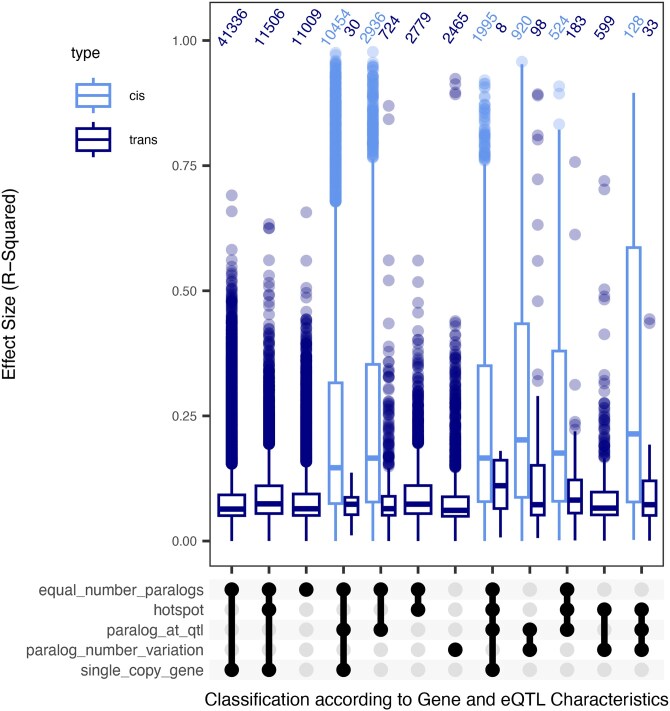
Distribution of QTL effect sizes according to genomic attributes of the transcript. Effect size of *cis* and *trans* eQTLs categorized according to the location of eQTL as well as paralog information of the transcript they control.

While a majority of *trans*-eQTLs are small-effect size, we did identify a number of large-effect *trans*-eQTL, and we wanted to understand what these may represent (Fig. 7). To better resolve these patterns and potential underlying mechanisms, we grouped all the eQTLs by their *cis*/*trans* status, presence in a hotspot, and presence of a paralog at the locus. One observation from this was that eQTLs with high-effect sizes often involve genes with multiple paralogs or copy number variation between accessions, especially those shared across multiple expression phenotypes. This agrees with previous observations that large-effect *cis*-eQTL are often associated with structural variation at the gene and these show large expression differences (Fig. 5; Li *et al.* 2013). The same pattern also held for large-effect *trans*-eQTLs where there was a paralog to the transcript mapping to the *trans*-eQTL. For example, the transcript AT1G2490 has a high-effect *trans*-eQTL across all 3 phenotypes, likely due to a paralog at the *trans-*eQTL that is present in Bay-0 but absent in Sha (Supplementary Table 1). Similarly, AT3G60980 has a high-effect *trans*-eQTL in SA and control, where Bay-0 retains an extra paralog at the *trans* position, but Sha has lost the copy (Supplementary Table 1).

Querying this list further identified genes where *cis* structural variation in the gene family at 1 locus links to a *trans* effect at the other members of the gene family. There are several possible mechanisms for this. First, it may be cross-recognition of the paralog by the microarray leading to a *trans* signal. Another option is that in the phosphoribosylanthranilate isomerase gene family in *Arabidopsis*, paralog variation at 1 locus creates a silencing mechanism decreasing expression of the other gene family members, as previously described (Bender 2004). Additionally, some genes are known to move laterally in the genome creating an appearance of a *trans* signal (West *et al.* 2006). There were a number of large-effect *trans*-eQTLs that have no connection to paralogues or paralogue variation (Fig. 7). This showed that *trans* hotspots contain large-effect eQTLs affecting a number of genes. These were distributed between *trans*-eQTLs that were both within and outside of genomic hotspots. Interestingly, the presence of large-effect *trans-*eQTLs within hotspots associated with plasticity suggests that pleiotropic *trans*-eQTLs are not obligated to have small effects. Assessing some of these genes showed that they included key components of the SA response and regulatory machinery (Supplementary Figs. 1 and 2; Supplementary Table 1). Thus, while the preponderance of effects at these loci is modest to small, there can be large *trans*-eQTLs that modulate the regulatory machinery directly influencing the plasticity.

## Discussion

Transcriptomic plasticity to SA treatment within the Bay × Sha-0 RIL population appears to be highly influenced by *trans*-regulatory eQTL. These plasticity eQTLs, because they are predominantly *trans*, tend to have a smaller effect than the *cis*-eQTLs (Supplementary Fig. 3). GWA analyses are typically underpowered to find small-effect loci, suggesting a potential for ascertainment bias in expression plasticity studies against these plasticity *trans*-eQTL. GWA is typically powered to identify moderate to larger effect loci, which in this case are *cis*-eQTL largely not associated with plasticity. This power bias could lead to an under-detection of *trans*-eQTL in GWA or other natural variation studies. Furthermore, previous studies have demonstrated that *trans*-regulatory variation can influence adaptive evolution within species (Rhoné *et al.* 2017; Lopez-Arboleda *et al.* 2021). For example in *Arabidopsis*, genes that show subpopulation-specific association are more likely to be exclusively *trans*-regulated (Lopez-Arboleda *et al.* 2021). This raises the potential that GWA studies have significant amounts of undetected *trans*-regulatory loci influencing gene expression and suggests that incorporating structured populations into plasticity studies will be key to better resolving the regulatory landscape of plasticity variation and helping to develop better models of how plasticity variation may evolve.

SA signaling is considered to be a highly conserved core component of plant responses to pathogen attack. This model was supported by previous studies identifying minimal variation in SA responses across *Arabidopsis* accessions (Koornneef *et al.* 2008). In agreement, we observed minimal variation in the SA transcriptome response between the Bay and Sha parents (Figs. 1 and 2). In contrast, the RIL progeny displayed extensive genotype × SA treatment variation across nearly the entire transcriptome, and this mapped to a defined set of *trans*-eQTL hotspots (Figs. 1 and 4). These hotspots pleiotropically influence the SA response of 100–1,000 s of transcripts (Fig. 4). The population size likely prevents any ability to see transgressive variation within *cis* variants due to the rarity of within-promoter recombination. It should be noted that the potential for *cis*-regulatory elements to generate transgressive phenotypes has been demonstrated in tomatoes with the use of CRISPR/Cas-9 technology, (Rodríguez-Leal *et al.* 2017). Because these hotspot loci had opposing directional effects on the response to SA, they created a situation whereby the parental accessions had largely the same SA response while the RIL progeny showed extensive transgressive segregation in SA response (Fig. 3). This shows that the minimal phenotypic plasticity to SA in *Arabidopsis* accessions hides genetic variation underlying the SA response, further supporting the need for structured populations in assessing plasticity variation within a species.

Previous research has observed that transgression is relatively common across plants and influences a diverse set of traits. The likelihood for transgressive segregation can increase for traits under constraining selection like a key defense response and also within selfing plant populations such as *Arabidopsis* (Rieseberg *et al.* 1999). Population structure limitations on gene flow can also create partitions that allow for the evolution of transgressive traits. Differentiating between these models would require the development of a number of structured populations across the species to better sample the potential for transgression to be a common aspect of typically assumed conserved signaling pathways. This is probably necessary to fully understand the genetic architecture of plasticity because extensive transgressive segregation across *Arabidopsis* accessions would influence the interpretation of GWA (Jimenez-Gomez *et al.* 2011). Transgressive variation and constraining selection would hide causal genetic variation that influences the trait and may be under locally adaptive selective pressures.

Observations from this structured population suggest that GWA studies of transcriptomic plasticity may need careful assessment. (1) Plasticity eQTL are biased toward small-effect *trans* loci which could go undetected in GWA studies. (2) Large-effect plasticity *trans*-eQTL can be linked to paralog variants, suggesting they are actually *cis* in causation. (3) Plasticity can display transgressive segregation that can constrain GWA ability to identify causal loci. While this is from a single structured population, it was randomly chosen with regard to the traits in question and the parents represent a broad sampling of *Arabidopsis* genetic diversity. While it will take additional structured populations to assess this potential more broadly, it does suggest there is a need to conduct these structured population studies more broadly to better understand the results from GWA collections.

## Supplementary Material

iyaf116_Supplementary_Data

## Data Availability

The microarray data set used in this study has been deposited at EBI ArrayExpress (http://www.ebi.ac.uk/arrayexpress/) under nos. E-TABM-126 and E-TABM-224. Detailed information on the *trans*-eQTLs with the highest effect sizes is found in Supplementary Table 1. Code for this research is maintained at https://github.com/mariele-lensink/plasticity_eQTL. Supplemental material available at GENETICS online.

## References

[iyaf116-B1] Ballinger MA, Mack KL, Durkin SM, Riddell EA, Nachman MW. 2023. Environmentally robust cis-regulatory changes underlie rapid climatic adaptation. Proc Natl Acad Sci U S A. 120(39):e2214614120. doi:10.1073/pnas.2214614120. 37725649 PMC10523592

[iyaf116-B2] Bender J . 2004. DNA methylation of the endogenous PAI genes in Arabidopsis. Cold Spring Harb Symp Quant Biol. 69(0):145–154. doi:10.1101/sqb.2004.69.145. 16117644

[iyaf116-B3] Broman KW, Gatti DM, Simecek P, Furlotte NA, Prins P, Sen Ś, Yandell BS, Churchill GA. 2019. R/qtl2: software for mapping quantitative trait loci with high-dimensional data and multiparent populations. Genetics. 211(2):495–502. doi:10.1534/genetics.118.301595. 30591514 PMC6366910

[iyaf116-B4] Brown KE, Kelly JK. 2022. Genome-wide association mapping of transcriptome variation in *Mimulus guttatus* indicates differing patterns of selection on cis- versus trans-acting mutations. Genetics. 220(1):iyab189. doi:10.1093/genetics/iyab189. 34791192 PMC8733635

[iyaf116-B5] Chan EKF, Rowe HC, Kliebenstein DJ. 2010. Understanding the evolution of defense metabolites in *Arabidopsis thaliana* using genome-wide association mapping. Genetics. 185(3):991–1007. doi:10.1534/genetics.109.108522. 19737743 PMC2907214

[iyaf116-B6] Dardiry M, Eberhard G, Witte H, Rödelsperger C, Lightfoot JW, Sommer RJ. 2023. Divergent combinations of cis-regulatory elements control the evolution of phenotypic plasticity. PLoS Biol. 21(8):e3002270. doi:10.1371/journal.pbio.3002270. 37590316 PMC10464979

[iyaf116-B7] de Nadal E, Ammerer G, Posas F. 2011. Controlling gene expression in response to stress. Nat Rev Genet. 12(12):833–845. doi:10.1038/nrg3055. 22048664

[iyaf116-B8] Ferrari S, Galletti R, Denoux C, De Lorenzo G, Ausubel FM, Dewdney J. 2007. Resistance to *Botrytis cinerea* induced in *Arabidopsis* by elicitors is independent of salicylic acid, ethylene, or jasmonate signaling but requires PHYTOALEXIN DEFICIENT3. Plant Physiol. 144(1):367–379. doi:10.1104/pp.107.095596. 17384165 PMC1913806

[iyaf116-B9] Fox J, Weisberg S. 2018. An R Companion to Applied Regression. Thousand Oaks (CA): Sage Publications.

[iyaf116-B10] Ghalambor CK, McKay JK, Carroll SP, Reznick DN. 2007. Adaptive versus non-adaptive phenotypic plasticity and the potential for contemporary adaptation in new environments. Funct Ecol. 21(3):394–407. doi:10.1111/j.1365-2435.2007.01283.x.

[iyaf116-B11] Gibson G . 2012. Rare and common variants: twenty arguments. Nat Rev Genet. 13(2):135–145. doi:10.1038/nrg3118. 22251874 PMC4408201

[iyaf116-B12] Göring HH, Terwilliger JD, Blangero J. 2001. Large upward bias in estimation of locus-specific effects from genomewide scans. Am J Hum Genet. 69(6):1357–1369. doi:10.1086/324471. 11593451 PMC1235546

[iyaf116-B13] He F, Arce AL, Schmitz G, Koornneef M, Novikova P, Beyer A, De Meaux J. 2016. The footprint of polygenic adaptation on stress–responsive cis-regulatory divergence in the Arabidopsis genus. Mol Biol Evol. 33(8):2088–2101. doi:10.1093/molbev/msw096. 27189540

[iyaf116-B14] He F, Steige KA, Kovacova V, Göbel U, Bouzid M, Keightley PD, Beyer A, De Meaux J. 2021. Cis-regulatory evolution spotlights species differences in the adaptive potential of gene expression plasticity. Nat Commun. 12(1):3376. doi:10.1038/s41467-021-23558-2. 34099660 PMC8184852

[iyaf116-B15] Hoekstra HE, Coyne JA. 2007. The locus of evolution: evo devo and the genetics of adaptation. Evolution. 61(5):995–1016. doi:10.1111/j.1558-5646.2007.00105.x. 17492956

[iyaf116-B16] Jimenez-Gomez JM, Corwin JA, Joseph B, Maloof JN, Kliebenstein DJ. 2011. Genomic analysis of QTLs and genes altering natural variation in stochastic noise. PLoS Genet. 7(9):e1002295. doi:10.1371/journal.pgen.1002295. 21980300 PMC3183082

[iyaf116-B17] Josephs EB, Stinchcombe JR, Wright SI. 2017. What can genome-wide association studies tell us about the evolutionary forces maintaining genetic variation for quantitative traits? New Phytol. 214(1):21–33. doi:10.1111/nph.14410. 28211582

[iyaf116-B18] Koornneef A, Leon-Reyes A, Ritsema T, Verhage A, Den Otter FC, Van Loon LC. 2008. Kinetics of salicylate-mediated suppression of jasmonate signaling reveal a role for redox modulation. Plant Physiol. 147(3):1358–1368. doi:10.1104/pp.108.121392. 18539774 PMC2442557

[iyaf116-B19] Korte A, Farlow A. 2013. The advantages and limitations of trait analysis with GWAS: a review. Plant Methods. 9(1):29. doi:10.1186/1746-4811-9-29. 23876160 PMC3750305

[iyaf116-B20] Lasky JR, Des Marais DL, Lowry DB, Povolotskaya I, McKay JK, Richards JH, Keitt TH, Juenger TE. 2014. Natural variation in abiotic stress responsive gene expression and local adaptation to climate in *Arabidopsis thaliana*. Mol Biol Evol. 31(9):2283–2296. doi:10.1093/molbev/msu170. 24850899 PMC4137704

[iyaf116-B21] Levis NA, Pfennig DW. 2016. Evaluating “plasticity-first” evolution in nature: key criteria and empirical approaches. Trends Ecol Evol. 31(7):563–574. doi:10.1016/j.tree.2016.03.012. 27067134

[iyaf116-B22] Li L, Petsch K, Shimizu R, Liu S, Xu WW, Ying K, Yu J, Scanlon MJ, Schnable PS, Timmermans MC, et al 2013. Mendelian and non-Mendelian regulation of gene expression in maize. PLoS Genet. 9(1):e1003202. doi:10.1371/journal.pgen.1003202. 23341782 PMC3547793

[iyaf116-B23] Li Y, Alvarez OA, Gutteling EW, Tijsterman M, Fu J, Riksen JA, Hazendonk E, Prins P, Plasterk RH, Jansen RC, et al 2006. Mapping determinants of gene expression plasticity by genetical genomics in *C. elegans*. PLoS Genet. 2(12):e222. doi:10.1371/journal.pgen.0020222. 17196041 PMC1756913

[iyaf116-B24] Lian Q, Huettel B, Walkemeier B, Mayjonade B, Lopez-Roques C, Gil L, Roux F, Schneeberger K, Mercier R. 2024. A pan-genome of 69 *Arabidopsis thaliana* accessions reveals a conserved genome structure throughout the global species range. Nat Genet. 56(5):982–991. doi:10.1038/s41588-024-01715-9. 38605175 PMC11096106

[iyaf116-B25] Liu X, Li YI, Pritchard JK. 2019. Trans effects on gene expression can drive omnigenic inheritance. Cell. 177(4):1022–1034.e6. doi:10.1016/j.cell.2019.04.014. 31051098 PMC6553491

[iyaf116-B26] Lopez-Arboleda WA, Reinert S, Nordborg M, Korte A. 2021. Global genetic heterogeneity in adaptive traits. Mol Biol Evol. 38(11):4822–4831. doi:10.1093/molbev/msab208. 34240182 PMC8557469

[iyaf116-B27] Loudet O, Chaillou S, Camilleri C, Bouchez D, Daniel-Vedele F. 2002. Bay-0 × Shahdara recombinant inbred line population: a powerful tool for the genetic dissection of complex traits in Arabidopsis. Theor Appl Genet. 104(6):1173–1184. doi:10.1007/s00122-001-0825-9. 12582628

[iyaf116-B28] Lovell JT, Sreedasyam A, Schranz ME, Wilson M, Carlson JW, Harkess A, Emms D, Goodstein DM, Schmutz J. 2022. GENESPACE tracks regions of interest and gene copy number variation across multiple genomes. Elife. 11:e78526. doi:10.7554/eLife.78526. 36083267 PMC9462846

[iyaf116-B29] Mai NT, Mai CD, Van Nguyen H, Le KQ, Duong LV, Tran TA, To HT. 2021. Discovery of new genetic determinants of morphological plasticity in rice roots and shoots under phosphate starvation using GWAS. J Plant Physiol. 257:153340. doi:10.1016/j.jplph.2020.153340. 33388665

[iyaf116-B30] Manners JM, Penninckx IA, Vermaere K, Kazan K, Brown RL, Morgan A, Maclean DJ, Curtis MD, Cammue BP, Broekaert WF. 1998. The promoter of the plant defensin gene PDF1.2 from Arabidopsis is systemically activated by fungal pathogens and responds to methyl jasmonate but not to salicylic acid. Plant Mol Biol. 38(6):1071–1080. doi:10.1023/A:1006070413843. 9869413

[iyaf116-B31] Marigorta UM, Rodríguez JA, Gibson G, Navarro A. 2018. Replicability and prediction: lessons and challenges from GWAS. Trends Genet. 34(7):504–517. doi:10.1016/j.tig.2018.03.005. 29716745 PMC6003860

[iyaf116-B32] Massouras A, Waszak SM, Albarca-Aguilera M, Hens K, Holcombe W, Ayroles JF, Dermitzakis ET, Stone EA, Jensen JD, Mackay TF, et al 2012. Genomic variation and its impact on gene expression in Drosophila melanogaster. PLoS Genet. 8(11):e1003055. doi:10.1371/journal.pgen.1003055. 23189034 PMC3499359

[iyaf116-B33] Metzger BPH, Wittkopp PJ, Coolon JD. 2017. Evolutionary dynamics of regulatory changes underlying gene expression divergence among Saccharomyces species. Genome Biol Evol. 9(4):843–854. doi:10.1093/gbe/evx035. 28338820 PMC5604594

[iyaf116-B34] Myles S, Peiffer J, Brown PJ, Ersoz ES, Zhang Z, Costich DE, Buckler ES. 2009. Association mapping: critical considerations shift from genotyping to experimental design. Plant Cell. 21(8):2194–2202. doi:10.1105/tpc.109.068437. 19654263 PMC2751942

[iyaf116-B35] Peng Y, Yang J, Li X, Zhang Y. 2021. Salicylic acid: biosynthesis and signaling. Annu Rev Plant Biol. 72(1):761–791. doi:10.1146/annurev-arplant-081320-092855. 33756096

[iyaf116-B36] Pigliucci M, Murren CJ, Schlichting CD. 2006. Phenotypic plasticity and evolution by genetic assimilation. J Exp Biol. 209(12):2362–2367. doi:10.1242/jeb.02070. 16731812

[iyaf116-B37] Pruunsild P, Bading H. 2019. Shaping the human brain: evolutionary cis-regulatory plasticity drives changes in synaptic activity-controlled adaptive gene expression. Curr Opin Neurobiol. 59:34–40. doi:10.1016/j.conb.2019.04.003. 31102862

[iyaf116-B38] Redman JC, Haas BJ, Tanimoto G, Town CD. 2004. Development and evaluation of an Arabidopsis whole genome Affymetrix probe array. Plant J. 38(3):545–561. doi:10.1111/j.1365-313X.2004.02061.x. 15086809

[iyaf116-B39] Rhoné B, Mariac C, Couderc M, Berthouly-Salazar C, Ousseini IS, Vigouroux Y. 2017. No excess of cis-regulatory variation associated with intraspecific selection in wild pearl millet (*Cenchrus americanus*). Genome Biol Evol. 9(2):388–397. doi:10.1093/gbe/evx004. 28137746 PMC5381623

[iyaf116-B40] Rieseberg LH, Archer MA, Wayne RK. 1999. Transgressive segregation, adaptation and speciation. Heredity (Edinb). 83(4):363–372. doi:10.1038/sj.hdy.6886170. 10583537

[iyaf116-B41] Rodríguez-Leal D, Lemmon ZH, Man J, Bartlett ME, Lippman ZB. 2017. Engineering quantitative trait variation for crop improvement by genome editing. Cell. 171(2):470–480.e8. doi:10.1016/j.cell.2017.08.030. 28919077

[iyaf116-B42] Schlichting CD, Smith H. 2002. Phenotypic plasticity: linking molecular mechanisms with evolutionary outcomes. Evol Ecol. 16(3):189–211. doi:10.1023/A:1019624425971.

[iyaf116-B43] Sham PC, Purcell SM. 2014. Statistical power and significance testing in large-scale genetic studies. Nat Rev Genet. 15(5):335–346. doi:10.1038/nrg3706. 24739678

[iyaf116-B44] Spoel SH, Dong X. 2024. Salicylic acid in plant immunity and beyond. Plant Cell. 36(5):1451–1464. doi:10.1093/plcell/koad329. 38163634 PMC11062473

[iyaf116-B45] Sun G, Yu H, Wang P, Lopez-Guerrero M, Mural RV, Mizero ON, Grzybowski M, Song B, Van Dijk K, Schachtman DP, et al 2023. A role for heritable transcriptomic variation in maize adaptation to temperate environments. Genome Biol. 24(1):55. doi:10.1186/s13059-023-02891-3. 36964601 PMC10037803

[iyaf116-B46] Takou M, Bellis ES, Lasky J. 2024. Predicting gene expression responses to environment in *Arabidopsis thaliana* using natural variation in DNA sequence. bioRxiv 591174. 10.1101/2024.04.25.591174, preprint: not peer reviewed. PMC12469744 41010053

[iyaf116-B47] Vande Zande P, Hill MS, Wittkopp PJ. 2022. Pleiotropic effects of trans-regulatory mutations on fitness and gene expression. Science. 377(6601):105–109. doi:10.1126/science.abj7185. 35771906 PMC9569154

[iyaf116-B48] Van Leeuwen H, Kliebenstein DJ, West MA, Kim K, Van Poecke R, Katagiri F, Michelmore RW, Doerge RW, St. Clair DA. 2007. Natural variation among *Arabidopsis thaliana* accessions for transcriptome response to exogenous salicylic acid. Plant Cell. 19(7):2099–2110. doi:10.1105/tpc.107.050641. 17630278 PMC1955704

[iyaf116-B49] Vlot AC, Dempsey DA, Klessig DF. 2009. Salicylic acid, a multifaceted hormone to combat disease. Annu Rev Phytopathol. 47(1):177–206. doi:10.1146/annurev.phyto.050908.135202. 19400653

[iyaf116-B50] Walter GM, Clark J, Terranova D, Cozzolino S, Cristaudo A, Hiscock SJ, Bridle J. 2023. Hidden genetic variation in plasticity provides the potential for rapid adaptation to novel environments. New Phytol. 239(1):374–387. doi:10.1111/nph.18744. 36651081

[iyaf116-B51] Wang D, Amornsiripanitch N, Dong X. 2006. A genomic approach to identify regulatory nodes in the transcriptional network of systemic acquired resistance in plants. PLoS Pathog. 2(11):e123. doi:10.1371/journal.ppat.0020123. 17096590 PMC1635530

[iyaf116-B52] West MA, Kim K, Kliebenstein DJ, Van Leeuwen H, Michelmore RW, Doerge RW, St. Clair DA. 2007. Global eQTL mapping reveals the complex genetic architecture of transcript-level variation in Arabidopsis. Genetics. 175(3):1441–1450. doi:10.1534/genetics.106.064972. 17179097 PMC1840073

[iyaf116-B53] West MA, van Leeuwen H, Kozik A, Kliebenstein DJ, Doerge RW, Clair DA, Michelmore RW. 2006. High-density haplotyping with microarray-based expression and single feature polymorphism markers in Arabidopsis. Genome Res. 16(6):787–795. doi:10.1101/gr.5011206. 16702412 PMC1473188

[iyaf116-B54] Xu C, Song LY, Zhou Y, Ma DN, Ding QS, Guo ZJ, Li J, Song SW, Zhang LD, Zheng HL. 2023. Integration of eQTL and GWAS analysis uncovers a genetic regulation of natural ionomic variation in Arabidopsis. Plant Cell Rep. 42(9):1473–1485. doi:10.1007/s00299-023-03042-5. 37516984

[iyaf116-B55] Zan Y, Shen X, Forsberg SKG, Carlborg Ö. 2016. Genetic regulation of transcriptional variation in natural *Arabidopsis thaliana* accessions. G3 (Bethesda). 6(8):2319–2328. doi:10.1534/g3.116.030874. 27226169 PMC4978887

[iyaf116-B56] Zhang X, Cal AJ, Borevitz JO. 2011. Genetic architecture of regulatory variation in *Arabidopsis thaliana*. Genome Res. 21(5):725–733. doi:10.1101/gr.115337.110. 21467266 PMC3083089

[iyaf116-B57] Zhang Y, Li X. 2019. Salicylic acid: biosynthesis, perception, and contributions to plant immunity. Curr Opin Plant Biol. 50:29–36. doi:10.1016/j.pbi.2019.02.004. 30901692

[iyaf116-B58] Zhao K, Aranzana MJ, Kim S, Lister C, Shindo C, Tang C, Toomajian C, Zheng H, Dean C, Marjoram P, et al 2007. An Arabidopsis example of association mapping in structured samples. PLoS Genet. 3(1):e4. doi:10.1371/journal.pgen.0030004. 17238287 PMC1779303

