# Supplementary material for: Trans-regulatory loci shape natural variation of gene expression plasticity in Arabidopsis
Source: Genetics. 2025 Jun 19;230(4):iyaf116. doi: 10.1093/genetics/iyaf116 (PMC12341950; doi:10.1093/genetics/iyaf116)
Supplement: iyaf116_Supplementary_Data [file iyaf116_supplementary_data.zip › Supplementary_Figure_Legends_GENETICS-2025-308129.docx]

**Supplementary Figure Legends**

**Supplementary Figure 1. Expression of transcripts with SA hotspot eQTLs on Chromosome 2 under salicylate and silwet treatment for parental and recombinant genotypes** The x axis is the genotype of RILS at the location of the SA and JA hotspots. For example, BB represents RILs with the Bay-0 genotype at both locations, where SB is RILs with the Sha genotype at the chromosome 2 hotspot and Bay-0 at the chromosome 5 hotspot.

**Supplementary Figure 2. Expression of transcripts with JA hotspot eQTLs on Chromosome 5 under salicylate and silwet treatment for parental and recombinant genotypes** The x axis is the genotype of RILS at the location of the SA and JA hotspots. For example, BB represents RILs with the Bay-0 genotype at both locations, where SB is RILs with the Sha genotype at the chromosome 2 hotspot and Bay-0 at the chromosome 5 hotspot.

**Supplementary Figure 3. Density distribution of cis and trans eQTL effect sizes.**

Effect size is represented as r-square value for cis (dark blue) and trans (light blue) eQTLs.
