# Supplementary figures and images for: Trans-regulatory loci shape natural variation of gene expression plasticity in Arabidopsis
Source: Genetics. 2025 Jun 19;230(4):iyaf116. doi: 10.1093/genetics/iyaf116 (PMC12341950; doi:10.1093/genetics/iyaf116)

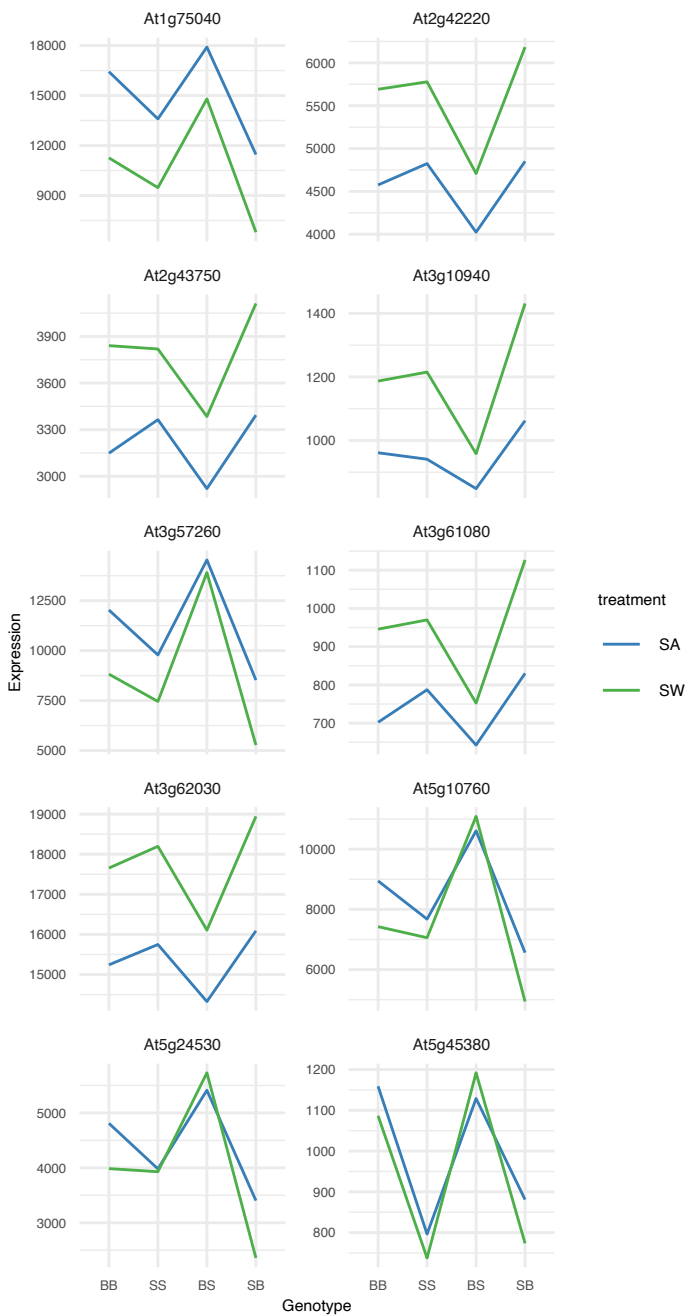

Supplement: iyaf116_Supplementary_Data [file iyaf116_supplementary_data.zip › Supplementary_Figure_1_GENETICS-2025-308129.pdf]

At1g74710

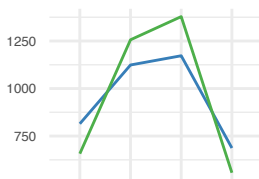

At2g04450

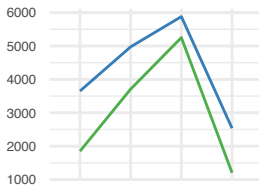

At3g52430

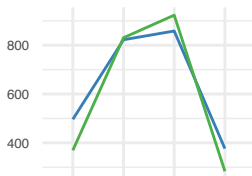

At3g60420

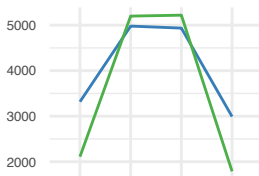

At4g12720

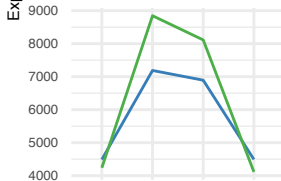

At4g39030

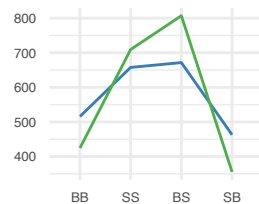

At5g61900

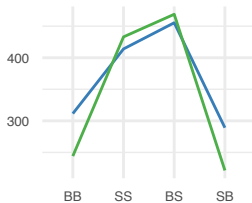

treatment

SA

SW

Genotype

Supplement: iyaf116_Supplementary_Data [file iyaf116_supplementary_data.zip › Supplementary_Figure_2_GENETICS-2025-308129.pdf]

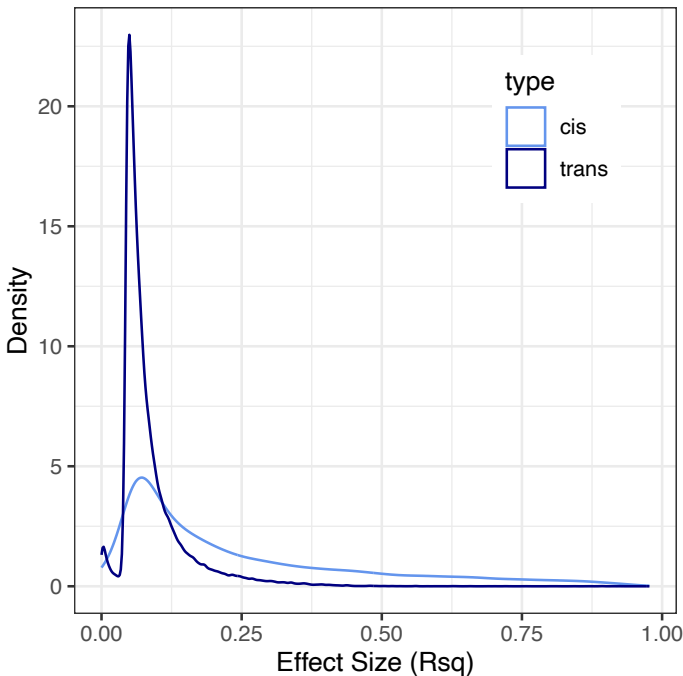

Supplement: iyaf116_Supplementary_Data [file iyaf116_supplementary_data.zip › Supplementary_Figure_3_GENETICS-2025-308129.pdf]
